# The role of an infection prevention and control nurse

**Published:** 2021-07-20

**Authors:** Jan West

**Affiliations:** 1 Former Senior Infection Prevention and Control Nurse, Royal Devon and Exeter NHS Foundation Trust, UK.

**A nurse may be assigned overall responsibility for infection prevention and control in an eye hospital or clinic – a demanding role that is vital for ensuring the safety of patients.**

**Figure:**
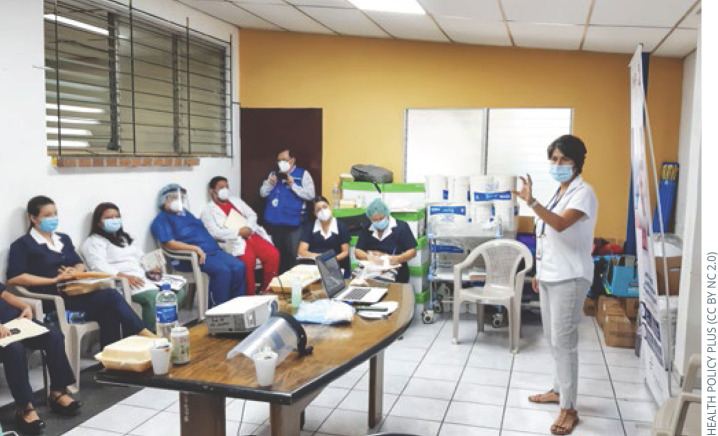
Infection control nurses are also responsible for training other staff members in infection control procedures. **EL SALVADOR**

Never has the role of infection prevention and control been more critical than during the current global COVID-19 pandemic.

Infection prevention and control involves many domains in the clinic or hospital setting, from patient management and staff health to environmental controls and building maintenance. The person assigned to lead infection prevention and control in an eye unit or hospital must therefore have a wide range of experience and may need to undertake additional training so they can give advice in a timely manner. There is also a need to be flexible and adapt guidelines to specific clinical situations. For example, the infection prevention and control nurse may be called upon to give decontamination advice on a new piece of equipment, so they must be able to review and evaluate evidence and critically appraise cleaning and disinfection products.

Quite often, there will be grey areas in infection prevention and control. The infection prevention and control nurse will be basing their advice on risk assessments, founded on a knowledge of the microorganisms involved, transmission routes, and work flow in the specialism. The advice may depend on the situation and how much risk the hospital, as an organisation, is willing to take. For example, in the event of an outbreak, it is possible to close a ward or department completely to new admissions, potentially stop all planned surgery, tests, and investigations, and restrict visiting, etc. Unfortunately, a lockdown also has some undesirable consequences, including patients not receiving a prompt diagnosis and treatment. Isolating a patient may also have negative consequences for those with mental health issues, which may need to be taken into account. Individual ethnic and cultural differences and requirements will also need to be considered – one topical example of this is the matter of facial hair (often mandated by a person’s religion or culture) and FFP3 mask fit testing.

Another essential attribute is to be both a good communicator and to be accessible, so that you can provide on-going support. There is no better way of becoming known, and to know what is *really* happening in clinical areas, than to visit and talk to people. It is surprising the conversations one may have about issues and queries that may never have been aired in a more formal setting.

**“An ability to review and evaluate evidence and critically appraise products is also essential.”**

There is also a need for a tenacious personality and honed negotiation skills – it can sometimes take months or years to persuade people to change their way of working, even when there is have good evidence to support a proposed measure.

The role suits people who are comfortable working with different people across a range of settings and are able to network and seek out expert opinion, whether by asking an appropriate individual or by carrying out internet and literature searches.

An ability to review and evaluate evidence and critically appraise products is also essential, as some products claim to work wonders, when in fact studies may not support this.

## Working environment

Depending on the size and nature of the eye service, the infection prevention and control nurse may be working alone; if this is the case, she or he would need to have sufficient knowledge of, and training in, infection prevention and control so that they can work unsupervised. Ideally, if resources allow, the infection prevention and control nurse should be supported by a team (see pp. 7). Support can also come from ‘clinical champions’ – doctors or nurses with an interest in infection prevention and control who can act as role models by demonstrating good practice. These clinical champions can also disseminate information to others, provide feedback about the existing programme and new proposals, and inform the infection prevention and control nurse about any concerns in their area of work.
